# Practice variation in induction of labour: women’s role in the decision-making process

**DOI:** 10.1007/s43999-025-00059-z

**Published:** 2025-02-19

**Authors:** Anne E. M. Brabers, Tamar M. Van Haaren–Ten Haken, Judit K. J. Keulen, Pien M. Offerhaus, Marianne J. Nieuwenhuijze, Judith D. de Jong

**Affiliations:** 1 Nivel (Netherlands Institute for Health Services Research), Utrecht, The Netherlands; 2 Research Centre for Midwifery Science, Zuyd University of Applied Sciences, Maastricht, The Netherlands; 3 Care and Public Health Research Institute, Maastricht University, Maastricht, The Netherlands

**Keywords:** Practice variation, Induction of labour, Maternity care, Women’s decision-making, Birth beliefs

## Abstract

In the Netherlands, percentages of induction of labour (IOL) range from 14.3 to 41.1% in regional maternity care networks (MCNs). In this study, we focus on women’s contribution in explaining this variation in range. We examine if different factors at the level of the individual woman (micro) and the level of the woman’s social context (meso) are related to decision-making on IOL, and the variation. We used an online questionnaire inviting women counselled for IOL (*n* = 180, response rate 40%) from six different MCNs, three with a high and three with a low percentage of IOL. Factors included are, for example, attitude towards birth, reason for IOL, and social norms. Descriptive statistics and regression analyses were performed to examine the relation between the included factors and the intended decision on IOL. Our results show that only the factor women’s attitude towards birth is related to the intended decision on IOL. The more women believe that birth is a medical process, the higher the odds that the intended decision is to induce labour. This may contribute to variation in IOL between individual women, but appears to contribute less to variation in IOL between MCNs. This is because the percentages of women with an intended decision for IOL do not differ within MCNs with a low or high percentage of IOL. A next step in explaining practice variation, is to examine mechanisms at the level of the individual healthcare provider (micro) and the MCN (meso).

## Background

Large practice variations in childbirth interventions have been observed within maternity care, both between and within countries [e.g. 1, 2, 3, 4]. A study comparing thirteen high-income countries showed large variations for interventions, such as augmentation of labour, induction of labour, pain relief, episiotomy, assisted vaginal birth, and caesarean section [2]. Practice variations in maternity care are not unique; the phenomenon of practice variation in medical treatment has been extensively described within the literature [e.g. 5, 6, 7]. Practice variation refers to “the degree to which healthcare providers differ in the frequency and/or way in which care is provided to patients with comparable care problems” [8; p. 17]. The underlying causes of practice variation are frequently unknown. Healthcare providers are often not able to clarify why practice variation exists. Policy makers often interpret variation as a signal of unnecessary care [9]. However, practice variation is not unambiguous ‘bad’ or ‘good’. The observation that there is practice variation does not directly provide information about the quality of care. Insight in the underlying causes of the variation is necessary to determine whether the observed variation is warranted or not. According to Wennberg [10], variation is only warranted when it can be explained by the patient’s health state or by patient’s preferences.

Also in the Netherlands, practice variation in maternity care is an issue that needs attention and requires further examination [11]. Dutch maternity care is organised regionally in maternity care networks (MCNs) (see Box 1 for more information about the Dutch maternity care system). Maternity care professionals, such as midwives, obstetricians and maternity nurses, work in close collaboration within these MCNs and are jointly responsible for providing high-quality maternity care in their region. In our multimethod VALID (VAriation in Labour InDuction) project, we examined practice variation between these MCNs in one specific intervention, namely induction of labour (IOL). We examined this for the so-called NTSV-group: women with a singleton pregnancy, who give birth to their *first* child in vertex presentation, after a gestation of at least 37 weeks (Nulliparous, Term, Singleton, Vertex). Using data from the years 2016–2018, we found large variation: the percentages of IOL within the NTSV-group ranged from 14.3 to 41.1% between the MCNs, with limited association to maternal outcomes and no association with perinatal outcomes [4]. Earlier research showed that mechanisms within MCNs (such as local protocols, and beliefs and attitudes towards childbirth) influence clinical decisions on interventions [12, 13]. It is important to get a deeper insight in the mechanisms that play a role in the decision-making on IOL, and how these mechanisms contribute to the observed practice variation between MCNs.

**Fig. 1:**
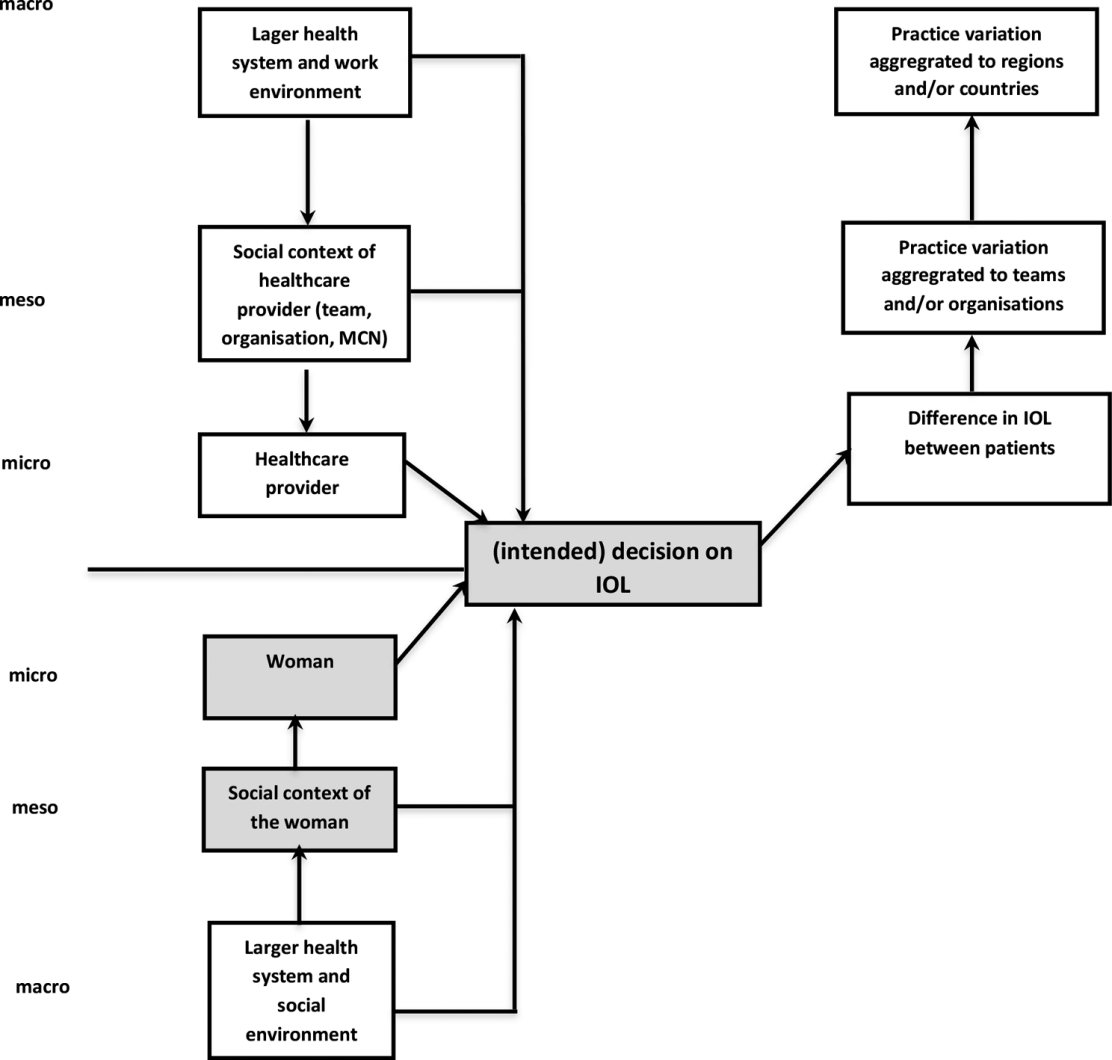
Sociological model explaining practice variation (based on [14, 15])

We use a sociological model explaining practice variation to get insight in the different mechanisms (see Fig. 1) [14, 15]. In this model three different levels (i.e. micro, meso and macro) are distinguished, at both the provider and the patient side. At each level, variation may be found and explanations for variation can be sought. In the current study, we focus on women’s role in the decision-making on IOL. An essential part of high-quality maternity care is a woman’s autonomy in the decision-making process, also when medical interventions are necessary [16]. In the Netherlands, decisions regarding medical interventions, like IOL, are preferably made by means of shared decision-making [17]. However, the ultimate decision-making responsibility lies with the woman [18].

At the level of the individual woman (micro), for example, attitudes/beliefs towards birth as a natural or medical process might be associated with the decision-making on IOL. While at the level of the woman’s social context (meso), existing norms in the woman’s social environment with regard to IOL may play a role. The aim of the current study is to examine different factors at both the level of the individual woman (micro) and the level of the woman’s social context (meso) (see grey blocks in Fig. 1) that might be related to a woman’s decision-making on IOL, and thus practice variation.

**Table:** 

**Box 1 Maternity care in the Netherlands** The majority of all Dutch women start antenatal care in primary midwife-led care in the community. They receive midwife-led care as long as pregnancy and birth develop normally, and they can opt for birth at home, in the hospital or birth center, supervised by their own midwife. In the situation of complications or risks, women are referred to secondary obstetrician-led care and birth will take place in the hospital. Interventions, like IOL, are only available in hospitals [19]. Midwifery practices and hospitals are organized in regional MCNs. Most of these MCNs are organized around one hospital and the midwifery practices in the region of the hospital. At the end of 2023, there were 70 MCNs [20]. The MCNs are responsible for providing high-quality maternity care in their region through collaboration between primary and secondary care providers.	

## Methods

This study has a cross-sectional study design using an online questionnaire.

### Setting

We use the MCNs as level of analysis in our VALID study. After calculating the percentage of IOL for all MCNs in the Netherlands separately, the MCNs were divided into four quartiles [4]. Subsequently, three MCNs from both Q1 (low rate of IOL) and Q4 (high rate of IOL) were recruited for the VALID study. The six MCNs are located in different regions of the Netherlands, including urban and rural areas. In both groups (Q1 and Q4), a MCN with an academic hospital was included.

### Participants

Within the six participating MCNs, women from the NTSV group who gave birth to their first child in the period October 2021 to July 2022 and who were counselled for a possible IOL were eligible to fill out the online questionnaire. This also includes women who decided not to be induced after counselling. The online questionnaire was open from January to October 2022. The women were recruited postpartum by the healthcare providers in the MCNs. If they gave informed consent, they received a link to the online questionnaire by e-mail from the research team. If necessary, we sent a reminder after two weeks. Finally, 180 women filled out the questionnaire, yielding a response rate of 40%. We have no information about the total number of eligible women during this period.

### Data collection

The questionnaire was developed by the authors, who have expertise on the research topic and in conducting survey-based research. The survey included, among others, questions about women’s background characteristics, attitudes, beliefs, preferences and experiences regarding childbirth, IOL and the decision-making process for IOL. If possible, validated questions or questions from earlier studies were used. A concept version of the questionnaire was tested with two women belonging to the NTSV group using the think aloud method. Based on their feedback, supplemented with feedback from a patient organization, we slightly adapted the formulation of several questions. No substantive changes were made.

### Variables / measurements

#### Intended decision on IOL

The dependent variable in this study is the intended decision on IOL, operationalized by the dichotomous question: “Has it been decided after discussions with your healthcare provider(s) to induce labour?” Not all women that had an intended decision for IOL were necessarily induced, as after the intended decision labour might have started spontaneously before the planned date of induction.

#### Factors on the micro and meso level of the woman

We used the Theory of Reasoned Action from Fishbein & Ajzen [21] as a theoretical framework. According to this theory, the intended decision for an IOL is influenced by women’s attitudes, social norms and perceived control as a result of personal, demographic, and environmental factors. We included the following factors on the micro level: ‘attitude’, ‘external necessity’, ‘information’, ‘timing’, and ‘involvement’. At the meso level we included ‘pressure/influence social environment (family and friends)’. Hereafter, we explain how each factor was operationalized in our study.

#### Attitudes

To measure the attitudes of women, we used the Birth Belief Scale (BBS) of Preis and Benyamini [22]. The BBS assesses basic beliefs about birth and consists of two subscales. One scale about birth as natural process (5 statements), and one scale about birth as medical process (6 statements). Respondents could answer on a 5-point Likert scale with scores ranging from totally disagree to totally agree. In the current study, the Cronbach’s alpha of the natural scale was 0.75, and of the medical scale 0.68. Both alphas were considered as reasonable. Scores for each subscale were derived by calculating the mean scores. A higher score indicates stronger beliefs about birth as a natural or medical process.

#### External necessity: reasons IOL

External necessity was operationalized as the reason to discuss IOL. We asked women for the main reason to discuss a possible IOL. Options were (with only one option possible): (1) long gestational age / over 41 weeks pregnant; (2) the pregnancy became too hard for me; (3) I wanted to plan the birth; (4) the baby moved little; (5) the baby was too small; (6) the baby was too big; (7) too little amniotic fluid; (8) the membranes ruptured without contractions; (9) I had high blood pressure / preeclampsia; (10) I had diabetes; 11) I don’t know, and 12) other reason. We recoded the answer options into three main categories: (1) prolonged pregnancy (option 1); (2) elective induction (options 2 and 3); and (3) medical reasons (options 4–10). The options 11 and 12 were recoded into missing (*n* = 26).

#### Information

Regarding the information women received about IOL, we asked the following question: Looking back, what did you think of the information you received about IOL? Women filled this in for three aspects, namely (1) clarity (2) sufficiency and (3) reliability, on a 5-point Likert scale ranging from 1, ‘totally disagree’ to 5, ‘totally agree’. To assess the coherence between the three aspects of information, the internal consistency of the three aspects was examined using Cronbach’s alpha. This showed an alpha of 0.91, which indicates good internal consistency. Because of this, a mean score was calculated over the three aspects ranging from 1 to 5.

#### Timing

This variable was measured using the question: At what gestational age did you discuss IOL for the first time? With options: 1) < 37 weeks; 2) at 37 weeks; 3) at 38–39 weeks; 4) around 40 weeks; 5) after 40 weeks; and 6) I don’t know. For the analysis, the answer options were recoded into three categories: (1) before/at 37 weeks, (2) at 38–39 weeks, and (3) around or after 40 weeks. The answer option I don’t know was recoded to missing (*n* = 6).

#### Involvement

We used the SDMQ-9 to measure the involvement of the woman in the decision-making on IOL. The SDMQ-9 is a validated instrument for measuring Shared Decision Making (SDM) [23]. We used the validated Dutch version of the SDMQ-9 [24], and adapted that version to the situation of IOL. E.g. ‘My doctor precisely explained the advantages and disadvantages of the treatment options’ was adapted to: ‘My midwife explained the pros and cons of both inducing labour and waiting’. When counselling only took place in a midwifery practice (n = 75), or the hospital (n = 35), a respondent filled out the SDMQ-9 once. When a respondent was counselled in both midwifery-led and in obstetrician-led care, she filled out the SDMQ-9 twice. In this last situation (n = 70), we used the scores of the SDMQ-9 of the healthcare professional in the hospital in the analyses, as the final decision for IOL was made in the hospital. All items are scored on six-point Likert scales ranging from 0 (completely disagree) to 5 (completely agree). In accordance with Kriston et al. [23], the sum scores were recalculated into a score on a 0-100 points scale, with higher scores meaning a greater degree of SDM.

#### Social environment

The social environment was measured by the perceived norms of the social environment regarding IOL. We included the descriptive norm. The descriptive norm was measured by the question: How often do you think labour is induced in women in your social environment? There were four answer options: (1) almost never; (2) sometimes; (3) often, and (4) almost always.

#### Background variables / confounders

Besides age (continuous) and educational level (in two categories: low (coded as 0) and high (coded as 1)), we included whether the woman received care in a MCN with a high rate of IOL (coded as 1) or in a MCN with a low rate of IOL (coded as 0) as a confounder.

### Statistical analyses

First, descriptive statistics were performed to get insight in the study population. Second, we tested the relation between each independent variable and the dependent variable (intended decision on IOL) using bivariate regression analyses. Third, we performed a multiple regression analysis in which we included all the factors that were statistically significant in the bivariate analyses. We controlled in all the regression analyses for the confounders age, educational level and MCN. In all the regression analyses, categorical variables were recoded into dummy variables. Furthermore, to make interpretation of the results more easy, we made sure that all variables in the regression analyses started at zero (e.g. age ranges from 21 to 42 years, but in the regression analyses from 0 to 21 years). The level of statistical significance was fixed at 0.05. All statistical analyses were carried out using Stata, version 16.

### Ethical considerations

The Medical Ethical Committee of Zuyderland-Zuyd University reviewed the proposal and confirmed that ethical approval was not required for the study according to the Dutch legislation and regulations (reference METCCZ20210008). All participants gave informed consent at the beginning of the online questionnaire.

## Results

In total, 180 respondents were included in the analyses (Table 1). The average age of the women was 31 years (range 21 to 42 years). Approximately two thirds (65%) of the women had a high educational level. More than half (55%) of the women had an intended decision to induce labour. Almost half of the women (47%) received maternity care within a MCN with a low percentage of IOL. According to half of the women (51%) a prolonged pregnancy was the reason to talk about IOL, and most discussions about IOL started for the first time in week 38–39 (39%).

**Table 1:** Descriptive statistics of the respondents

	Total (*N* = 180)*	Intended decision on IOL – No (*N* = 81)	Intended decision on IOL – Yes (*N* = 99)
**Age (mean**,** std dev)**	31 (3.8) Range 21–42	32 (3.6) Range 23–42	31 (3.9) Range 21–42
**Educational level**			
Low/Middle (none, primary school or pre-vocational education, and secondary or vocational education)	35%	32%	37%
High (professional higher education or university)	65%	68%	63%
**MCN**			
Low % of IOL	47%	51%	43%
High % of IOL	53%	49%	57%
**Intended decision on IOL**			
No	45%	Not applicable	Not applicable
Yes	55%		
**Attitudes**			
BBS – medical	3.4 (0.59) Range 1.7–4.7	3.2 (0.56) Range 1.7–4.5	3.5 (0.56) Range 2.2–4.7
BBS – natural	3.9 (0.59) Range 2.2-5	4.1 (0.53) Range 2.8-5	3.8 (0.60) Range 2.2-5
**External necessity: reasons for discussion on IOL**	(*N* = 154)**	(*N* = 65)**	(*N* = 89)**
Prolonged pregnancy	51%	65%	42%
Elective induction	13%	8%	17%
Medical reason	36%	28%	42%
**Information** (mean score 3 aspects: clarity, sufficiency and reliability) (range 1–5)	4.0 (0.90) Range 1–5	4.1 (0.85) Range 1.7-5	3.9 (0.93) Range 1–5
**Timing**	(*N* = 174)***	(*N* = 75)***	(*N* = 99)***
before/at 37 weeks	29%	28%	30%
at 38–39 weeks	39%	44%	34%
around or after 40 weeks	32%	28%	35%
**Involvement decision-making (mean, std dev)**			
SDMQ-9 score (range 0-100)	69.1 (21.3) Range 8.9–100	71.7 (19.6) Range 8.9–100	67.0 (22.4) Range 20–100
**Social environment** (Descriptive norm: how often IOL in social environment?)			
Almost never	10%	12%	8%
Sometimes	57%	62%	54%
Often/almost always****	33%	26%	38%

* If less than 180 respondents filled out the question, the N is given in the Table. The percentages are then calculated for the respondents that filled out the question

** 26 women were recoded to missing for this variable as they answered I don’t know or other reason on this question

*** 6 women were recoded to missing for this variable as they answered I don’t know on this question

****Often and almost always are taken together as only 2 out of the 180 women filled out almost always

Tables 2a and 2b give an overview of the bivariate analyses between each factor and the intended decision on IOL (controlled for age, educational level, and the level of IOL in MCN). The results show that the factor ‘attitudes’ (model I) is significantly related to the intended decision on IOL, as well as the factor ‘external necessity’ (model II). For the other four factors (model III, IV, V, and VI) no significant relationship with the intended decision on IOL was found. The results show that the higher women score on the medical subscale of the BBS, the higher the odds that the intended decision was to induce labour (OR: 2.26; 95% CI: 1.21–4.21). The results also show that the higher women score on the natural subscale of the BBS, the lower the odds that the intended decision was to induce labour (OR: 0.47; 95% CI: 0.25–0.88). With regards to the factor ‘external necessity’, we found that women for whom the main reason to discuss a possible IOL belong to the category elective induction (OR: 3.57; 95% CI: 1.14–11.15) or medical reasons (OR: 2.26; 95% CI: 1.06–4.81) had a higher odds that the intended decision was to induce labour than women for whom the main reason to discuss a possible IOL was a prolonged pregnancy. In addition, we observed in model II and IV a significant relationship between age and the intended decision for IOL. The older the woman, the lower the odds that the intended decision was to induce labour.

**Table 2a:** Results of the bivariate analyses – models I, II, and III

Intended decision on IOL (1 = yes, 0 = no)	Model I Relation attitudes and intended decision on IOL (*n* = 180)		Model II Relation external necessity and intended decision on IOL (*n* = 154)		Model III Relation information and intended decision on IOL (*n* = 180)	
	OR (95% CI)	*p*-value	OR (95% CI)	*p*-value	OR (95% CI)	*p*-value
**Age (continuous)**	0.93 (0.85–1.01)	0.097	0.90 (0.81–0.99)	**0.035**	0.92 (0.85–1.01)	0.072
**Educational level (0 = low/middle**,** 1 = high)**	1.26 (0.62–2.55)	0.530	1.68 (0.77–3.68)	0.194	1.01 (0.52–1.98)	0.973
**MCN (0 = low**,** 1 = high)**	1.21 (0.62–2.34)	0.573	1.38 (0.69–2.75)	0.364	1.19 (0.64–2.22)	0.578
**Attitudes**						
BBS – medical	2.26 (1.21–4.21)	**0.010**				
BBS – natural	0.47 (0.25–0.88)	**0.018**				
**External necessity: reasons IOL**						
Prolonged pregnancy			Reference			
Elective induction			3.57 (1.14–11.15)	**0.029**		
Medical reason			2.26 (1.06–4.81)	**0.034**		
**Information**					0.74 (0.53–1.06)	0.103
**Timing**						
before/at 37 weeks						
at 38–39 weeks						
around/after 40 weeks						
**Involvement decision-making (SDMQ9 score**,** 0-100)**						
**Social environment (descriptive norm)**						
Almost never						
Sometimes						
Often/almost always						
**Constant**	1.95	0.526	1.63	0.426	6.05	**0.023**

**Table 2b:** Results of the bivariate analyses – models IV, V, and VI

Intended decision on IOL (1 = yes, 0 = no)	Model IV Relation timing and intended decision on IOL (*n* = 174)		Model V Relation involvement and intended decision on IOL (*n* = 180)		Model VI Relation social environment and intended decision on IOL (*n* = 180)	
	OR (95% CI)	*p*-value	OR (95% CI)	*p*-value	OR (95% CI)	*p*-value
**Age (continuous)**	0.91 (0.84-1.00)	**0.045**	0.92 (0.85–1.01)	0.080	0.93 (0.85–1.02)	0.119
**Educational level (0 = low/middle**,** 1 = high)**	1.10 (0.55–2.19)	0.785	0.96 (0.49–1.87)	0.898	0.96 (0.49–1.8)	0.896
**MCN (0 = low**,** 1 = high)**	1.23 (0.65–2.32)	0.525	1.13 (0.61–2.11)	0.690	1.06 (0.57-2.00)	0.846
**Attitudes**						
BBS – medical						
BBS – natural						
**External necessity: reasons IOL**						
Prolonged pregnancy						
Elective induction						
Medical reason						
**Information**						
**Timing**						
before/at 37 weeks	Reference					
at 38–39 weeks	0.74 (0.35–1.58)	0.437				
around/after 40 weeks	1.27 (0.57–2.84)	0.553				
**Involvement decision-making (SDMQ9 score**,** 0-100)**			0.99 (0.97-1.00)	0.114		
**Social environment (descriptive norm)**						
Almost never					Reference	
Sometimes					1.37 (0.49–3.81)	0.543
Often/almost always					2.17 (0.73–6.48)	0.165
**Constant**	2.91	0.071	5.81	**0.024**	1.60	0.491

Subsequently, we performed a multivariate logistic regression analysis in which we included both the factors ‘attitudes’ and ‘external necessity’ as independent variables. Table 3 shows that in this model only one aspect of the factor ‘attitude’ is significantly related to the intended decision on IOL. The more women believe that birth is a medical process, the higher the odds that the intended decision was to induce labour (OR: 2.08; 95% CI: 1.05–4.12). In addition, we also found in this model that the older the woman, the lower the odds that the intended decision was to induce labour.

**Table 3:** Results of the multivariate logistic regression analysis – model VII

Intended decision on IOL (1 = yes, 0 = no)	Model VII Relation attitudes and external necessity and intended decision on IOL (*n* = 154)	
	OR (95% CI)	*p*-value
**Age (continuous)**	0.90 (0.81–0.99)	**0.039**
**Educational level (0 = low/middle**,** 1 = high)**	1.97 (0.87–4.47)	0.106
**MCN (0 = low**,** 1 = high)**	1.36 (0.66–2.83)	0.406
**Attitudes**		
BBS – medical	2.08 (1.05–4.12)	**0.036**
BBS – natural	0.61 (0.31–1.21)	0.156
**External necessity: reasons IOL**		
Prolonged pregnancy	Reference	
Elective induction	2.79 (0.87–8.96)	0.085
Medical reason	1.89 (0.85–4.19)	0.119
**Constant**	1.10	0.935

## Discussion

Our results show that, at the micro level, the attitude of women regarding birth is related to the intended decision on IOL. The higher women score on the medical subscale of the BBS, the higher the odds that the intended decision is to induce labour. For none of the other included factors, at both the micro and the meso level, we found a relationship with the intended decision on IOL in the multivariate analysis. Besides, we found that the older the woman is, the lower the odds that the intended decision is to induce labour. Although our findings may explain some of the variation between individual women, it does not explain variation between MCNs.

Our finding that the attitudes of women regarding birth plays a role in the intention to IOL, is in line with earlier research. For example, Preis et al. [25] found that stronger beliefs about birth as a medical process were related to fewer natural birth choices. In addition, the more women believed birth to be a natural process, the less they opted for medical birth-related choices, like the induction of labour. Next to this, Haines et al. [26] found an association between the attitude and the type of birth. In their study, women belonging to the so-called ‘self-determined’ cluster, and seeing birth as a natural process, had the highest percentage of unassisted vaginal births [26]. Contrary, women belonging to the so-called ‘fearful’ cluster, who did not see birth as a natural event, had a greater likelihood of having an elective caesarean. Halfdansdottir et al. [27] found that women having a positive attitude towards home birth had significantly more positive attitudes towards birth and more negative attitudes towards interventions. The study of Keulen et al. [28] observed that the main reason for women to prefer expectant management at 41 weeks was their wish to give birth as natural as possible.

Women’s attitudes, and the subsequent decision-making regarding IOL, might be influenced by from whom and where, they receive care. For example, earlier research among healthy nulliparous women showed that women using midwife-led care reported attitudes supporting less frequent use of technology compared to women receiving care from obstetricians [29]. Akuamoah-Boateng and Spencer [30] conclude, in their systematic review of qualitative evidence, that women’s decision making about IOL is influenced by, among others, healthcare professionals. Also the information women receive has an impact on their choice and decision-making about IOL [30]. In our study, the included women received care in six different MCNs, of which three have a high percentage of IOL and three a low. Each of these MCNs often have their own local protocols, and beliefs and attitudes regarding IOL. It can be expected that these mechanisms are related to the care and information provided to women, and subsequently to their attitude, and (intended) decision on IOL. In our study, we found a small difference in the percentage of women with an intended decision for IOL between MCNs with a high and low percentage of IOL: 58% of the women in MCNs with a high percentage had an intended decision for IOL, compared to 51% of the women in MCNs with a low percentage of IOL (not in table, overall percentage that had an intended decision on IOL 55%). However, this tendency does not differ significantly (*p* = 0.337, not in table, and see Table 2a, 2b and 3). For future research, it is recommended to get a better insight in the role of the MCN in the decision-making on IOL, as individual differences between women appear not to explain variation between MCN’s.

We also found a relationship between age and the intended decision on IOL: the older the women, the lower the odds that the intended decision is to induce labour. This is in agreement with earlier research. Keulen et al. [28], for example, observed that women who had a preference for IOL were on average younger than women who preferred expectant management.

In the first step of the VALID study we observed high practice variation in IOL between MCNs in the relatively homogenous NTSV group. We argued that it is unlikely that this variation can be fully explained by pregnancy complications like pregnancy hypertension [4]. We therefore aimed to explain this variation using a sociological model of practice variation. In this study, we hypothesized that both mechanisms at the micro and the meso level of the women play a role in the decision-making process. At the micro level, we found that one mechanism, namely the attitude towards birth, plays a role in the decision-making process on IOL. However, at the meso level, we did not find that the mechanism of social norms was related to the intended decision for IOL. It appears that the intended decision for IOL is mainly associated with personal, intrinsic factors, such as attitudes, and less with social norms. It is not clear to what extent social norms and environmental factors influence the shaping of women’s attitudes towards birth.

Our results also show that the attitude of women contributes to practice variation in IOL between individual women, but to a lesser extent to practice variation in IOL between MCNs. The reason for this is the observation that we do not find an association whether a woman receives care in a MCN with a low or high percentage of IOL and the intended decision for IOL. This suggests that primarily mechanisms at the provider side of our sociological model explain the high practice variation in IOL between MCNs, either at the micro level of the individual healthcare provider, or at the meso level of the MCN. Possible mechanisms might be regional guidelines and protocols, the social norms in MCNs or the preferences of providers regarding IOL. Currently, within the VALID study, several other studies are performed to gain insight into this, such as document analyses of available protocols and guidelines, a survey and interviews with providers. The last step will be to wrap up all the results of the separate studies in an expert meeting to get insight in which mechanisms contribute to the observed variation, and which of these result in unwarranted variation.

### Strengths and limitations

A strength of this study is that our starting point was a sociological model including several levels at which variation may be found and where explanations can be sought. Another strength is that we used, if available, validated scales (e.g. the SDMQ-9 and the Birth Believe Scale) to measure the concepts included in this study. Another strong point is that we included women from both MCNs with a low and high percentage of IOL. Unfortunately, we were not able to analyse the results for each MCN separately due to the low number of women per MCN. Furthermore, the women were recruited personally by the healthcare providers within the MCNs. This might have resulted in a selection-bias as we have no information about the total group of eligible women. Another limitation is that we measured all our concepts after birth, including the attitude towards birth. It might be that women’s attitude towards birth is influenced by the experiences women had during birth.

## Conclusions

Our results show that the attitude towards birth is related to the intended decision on IOL. The more women believe that birth is a medical process, the higher the odds that the intended decision is to induce labour. This result may influence practice variation in IOL between individual women, but it may contribute to a lesser extent to practice variation in IOL between MCNs. This is because the intended decision does not differ for women receiving care in a MCN with a low or high percentage of IOL. A next step is to examine mechanisms at the level of the individual healthcare provider (micro) and the MCN (meso) to get insight in whether explanations for variation in IOL can be found at these levels.

## Data Availability

Data are available upon reasonable request from the corresponding author.
